# Imbalanced Diet Deficient in Calcium and Vitamin D- Induced Juvenile Osteopenia in Rats; the Potential Therapeutic Effect of Egyptian Moghat Roots Water Extract (*Glossostemon bruguieri*)

**Published:** 2014

**Authors:** Doaa A Ghareeb, Fatma H El-Rashidy, Sherif El-Mallawany

**Affiliations:** a *Department of Biochemistry, Faculty of Science, Alexandria University, Alexandria, Egypt.*; b *Department of Chemistry, Faculty of Science, Alexandria University, Alexandria, Egypt. *

**Keywords:** Juvenile osteopenia, Synthetic diet for rat, *Glossostemon bruguieri*, Rat PTH, Rat estrogen

## Abstract

This study aimed to explore and validate a new juvenile osteopenic (JO) rat model then examine the efficacy of moghat (*Glossostemon bruguieri*) as an alternative reversal therapy for JO. Phytochemical screening analysis showed that moghat contains 5.8% alkaloids, 1.5% flavonoids and 13.2% total phenols. Juvenile osteopenia was induced in 15 days old Sprague- Dawley female rats by feeding them free Ca and vitamin D synthetic diet for 21 days. Osteopenic rats were either treated with moghat (0.8 g dried plant tissue/Kg body weight, orally), or with a reference nutritional supplements of calcium chloride (14 mg Ca/Kg) and vitamin D3 (7 IU/Kg), for extra 21 days. Both untreated and treated groups were compared to a control group that fed a regular pelleted food. Our results showed that osteopenic rats lost normal bone tissue architecture, 30 % of body mass, 54 % of bone mass and finally 93% of bone calcium mass. Furthermore, these rats showed a markedly increase in serum phosphate, PTH, alkaline phosphatase, aspartate transaminase activities and creatinine level as compared to the control group. Moghat administration was successfully reversed osteopenia by normalizing body and bone masses to the reference ranges, increased the bone calcium mass by 17 fold without any detectable side effects on liver and kidney physiological performance. Therefore, moghat could be considered as potent safe –JO- reversal extract.

## Introduction

Osteopenia is a skeletal disorder characterized by low bone mass and micro-architectural deterioration of bone tissue with a consequent increase in bone fragility and susceptibility to fracture risk. Osteopenia in children is rare and mostly is a secondary concomitant to conditions such as prolonged immobilization, malabsorption syndromes, corticosteroid excess, osteogenesis imperfecta, Celiac disease, Turner’s syndrome, malignancy and homocystinuria. After the exclusion of decreased bone mass causes, one form of osteopenia remains over which is idiopathic juvenile osteopenia (IJO) that was firstly described by Schippers in 1938.

IJO is a sporadic disorder of children manifesting in healthy prepubertal children between the 2^nd^ and 13^th^ year of the life, however very young patients who are 9 months, 1.5 years and 2.0 years of age with IJO have also been reported (1). The typical IJO symptoms include walking difficulty, with a characteristic gait and pain in the back, hips and feet (1). 

Most patients might be recovered throughout the first 2 to 4 years of juvenile osteopenia (JO) initial manifestation. Furthermore, such disease progression could be spontaneously arrested during or even after puberty. Only few adult patients with severe conditions of marked skeletal deformities may remain disabled (2).

Throughout the life, our choices are affecting the bone mass (BMC) and density (BMD) where the physical activity level, the amount of meal calcium and other food ingredients are accounting as important factors that influence the development of peak bone mass and the rate at which bone is lost later in life (3). Diet deficient in vitamin D_3_ and/or calcium may lead to the development of rickets in children and osteomalacia in adults (4). Sodium accounts as important ions that plays an essential role in BMC, because it locates superficially in bone tissue. There are statistically significant positive and quite consistent correlations between 24 h urine sodium and 24 h urine calcium excretion. It is reported that urine calcium rises from 0.5 to 1.5 mmol (20–60 mg) for every 100 mmol (2300 mg) sodium ingested, therefore high sodium intake leads to calciuria. Thus, on prevailing diets, sodium intake accounts for much of the obligatory calcium urinary loss from the body, and for this reason, it would be play a crucial role in the osteoporosis pathogenesis (5).

Despite protein is very important ingredient for human diet, high protein intake, especially those rich in animal protein, is reported as a determinant factor for urinary calcium excretion and thus it is inadvisable for long-term bone health (6).

The challenge that faced the scientists who worked in JO area is how to produce a valid animal model to track this disease, which in turn, give a chance to examine and develop new preventive and curative strategies. So the first motivation in this study was to establish a new JO animal model by using a simulator human unhealthy –imbalanced- poverty food ingredient. 

This diet of imbalanced calcium- phosphorus ratio contributed high sodium level and calcium deficient protein, egg albumin instead of casein. These ingredients when collected in one diet accounted as insurance to osteopenia incidence.

Several regiments for general osteopenia treatment were established several years ago but the efficacy of these regiments is very low or takes several years to improve the case. The modern therapy recommended for the treatment of osteopenia includes supplementation with estrogen, progestin, calcitonin, bisphosphonates, *etc.* But these therapeutic managements possess several down-sides and available evidence suggests that long-term use of estrogen replacement therapy as example may have serious side effects such as breast or uterus cancer (7). Moreover, there is no established medical or surgical therapy for JO. However, physical therapy, using crutches, avoiding unsafe weight-bearing activities, and other supportive care are the first step used to treat JO, along with well-balanced diet rich in calcium and vitamin D (8). 

For that, scientists throughout the world are looking for better alternative therapeutic management, especially from natural resources which are thought to be healthier and safer for the treatment of osteopenia (7).

Moghat, *Glossostemon bruguieri* belongs to the Family *Buttneriaceae* (*Sterculaceae*). The plant grows wild in Iraq and Iran from where it was imported. It was introduced to Egypt in 1932 and continued to be cultivated in very small area (9).

Moghat is accounting as an amazing food supplement where its root dry matter contains 24% starch, 5% pectin, 3% sugars, 5% fats, 5.5% total crude protein, 23% mucilage on basis of dry matter and mineral elements. Methionine, tyrosine, proline, threonine, glutamic acid, glycine, serine, argenine and aspartic acid have been identified in the roots, glutamic and aspartic acids being the major ones. The powdered roots with some additives, spices, flavoring agents, sugars and butter, are used by the majority of Arab nations for preparing a hot drink especially in winter, and to stimulate lactation in nursing mothers (10).

Moghat root is used in Folk medicine as tonic to increase the body weight and for the treatment of bruises, gout and spasms. It is also considered to be a demulcent (11). Furthermore, it has had remarkable hypoglycemic activity as moghat intake was decreasing the blood glucose levels in diabetic rats by 54.5% within 15 days (12).

Moghat have had oestrone, scopoletin, phytosterols (a mixture of β-sitosterol, stigmasterol and campesterol). In 2003, Meselhy (12) isolated biflavonemoghatin, together with five known compounds: 4′-methoxyisoscutellargin, sesamin, chrysophanol, emodin and methoxyemodin (physcion). These constituents exhibit a variety of biological effects such as antioxidant and anti-diabetic (11, 12). This broad spectrum of constituents suggests the possible utilization of Moghat as a valuable crude alternative therapy (11, 12).

Isoflavone is naturally occurring substance such as genistein (isoflavone isolated from soybeans) that has estrogenic-like activity, which binds to osteoblastic estrogen receptors and plays an unique role in bone mass maintenance in ovariectomized rats (13). Therefore, isoflavone provides a rationale for its use in the prevention and treatment of osteopenia syndromes (14). 

As moghat contains phytoestrogen; such as isoflavone (biflavone), so it could be used as an osteopenia preventer. This study was aimed to explore and validate a new juvenile osteopenic (JO) rat model then examine the efficacy of Moghat as alternative therapy to reverse JO in this model in comparison to reference nutritional calcium and vitamin D supplement, the first therapeutic regimen used in JO treatment. Indeed the use of this reference drug gave the chance to deduce if the treatment with calcium and vitamin D combination reversed the detriment effect of synthetic imbalanced diet on bone tissue. 

## Experimental

*Materials*

Calcium chloride and Vitamin D3 were purchased from Sigma Chemical Company (St. Louis, Mo, USA). All other vitamins were bought from RP Scherer, Cairo, Egypt. Commercial kits for AST, ALT, ALP, creatinine and urea were bought from Randox Company (France). All other chemicals and reagents of highest quality were available commercially. Moghat (*Glossostemon bruguieri*) was purchased from Egyptian local market and authenticated by Prof. Dr. Eldareir, S. Prof. of plant ecology, Faculty of Science, University of Alexandria, Egypt. 

*Animals*

All animal experiments were performed according to the Guide for the Care and Use of Laboratory Animals, National Institute of Health (15). One hundred and thirty four female Sprague Dawley rats aged 7- 90 days-old for estrogen level determination and osteopenia induction/ treatment and 104 albino mice aged 60 days- old for moghat acute and chronic toxicity determination were obtained from the experimental animal house, Faculty of Medicine, University of Alexandria. Sprague Dawley rats were divided as follow; eighty four different age normal female rats were used to determine the puberty onset age, while 50 rats aged 15 days used in JO induction and treatment protocols. The animals were grouped in metal cages (5 rats/cage, maintained at approximately 23-25 °C with a 12 h light/dark cycle and received basal diet and tap water *ad-libitum* for one week acclimation period.

*Phytochemical screening of moghat*

Qualitative chemical tests for tannins, phlobatannins, saponin, flavonoids, steroids, terpenoids and cardiac glycosides as well as quantitative determination of the total phenols, alkaloid and flavonoid were carried out on the aqueous moghat extract and on the powdered specimens using standard procedures as described by Edeoga *et al.* (16). While steroid compounds was determined by using estrogen ELISA kit. Furthermore, the moghat calcium content was estimated in root ash using atomic absorption spectrophotometer (Perkin-Elmer, Germany) (17).

*Moghat suspension preparation*

Moghat root was grinded into powder form, then soaked in boiled sterilized distilled water at a concentration of 24 mg/mL and kept in 4 ^ᵒ^C. Each couple of days, a newly suspension was performed.

*Acute and sub-chronic toxicity study*

The acute and chronic toxicity study was performed as per the method described by Litchfield and Wilcoxon (1949), and LD_50_ was calculated accordingly. For acute toxicity, aqueous suspension of moghat in the dose range of 100–2000 mg/Kg was orally administered to different groups of mice (n = 6). The animals were examined at every 30 min up to a period of 3 h and then occasionally for additional 4 h period, finally 24 h mortality was recorded. For sub-chronic toxicity or extract safety determination, four mice group (n=10) was orally administrated with 0.2 mL of aqueous suspension of moghat at concentration of (200, 500, 1000 or 2000 mg/Kg) for one month. Liver and kidney function were determined in mice sera and comparing with control group that received 0.2 mL water.

The antiosteoporotic activity was performed on female Sprague–Dawley rats at dose of 800 mg/Kg of body weight.

*Level of blood estrogen as a function of age*

Fourteen groups (6 rats/ group) of normal female rats aged from 7 to 98 days old (the age difference between groups was 7 days old) were fasted overnight, decapitated, then their sera were collected and stored at -20 ^o^C for estrogen analysis. 

*Free calcium and vitamin D – synthetic – diet *was prepared according to Ghareeb *et al.*, (18) as mentioned in Table 1. The diet was baked at 150 ^ᵒ^C for 90 min. then grinded into pellet form. Each 100 g diet provided the animals with 1784 k joule or 420 kcal. 

**Table 1:** The synthetic diet ingredients

**Component**	**JO diet**
Egg albumin	12
Corn starch	50
Sucrose	19
Cellulose	5
Mineral mixture	4*
Corn oil	9
Vitamin mix	1**

* This mixture contained 0.18 g sucrose instead of 0.18 g CaCl_2_.

** This mixture lack 60IU (1.5 µg) vitamin D_3_.

*JO induction*

JO was induced by feeding forty rats (15 days old) the free calcium and vitamin D synthetic diet for 21 days (100 g/Kg). After this induction period, ten rats were scarified (induced-untreated 21) and the other 30 rats were divided into three subgroups, all subgroups were kept on the same synthetic diet, as follow; 1) 10 rats were orally administrated 0.5 mL distilled water (induced- untreated 42), 2) another 10 rats were orally treated with a 0.5 mL of water suspension of powdered moghat at dose of 0.8 g/Kg, 3) and the last 10 rats were orally administrated with 0.5 mL reference nutritional supplement which containing 0.75 mM CaCl_2_ and 2 IU Vitamin D3. All treated rats were compared to a control group (10 rats), which were fed a regular pelleted diet and allowed for free access to tap water. 

At the end of experiment period, rats in all groups were fasted overnight and then decapitated. Blood was collected and sera were separated off to estimate estrogen level, parathyroid hormone (PTH), calcium and phosphate levels. Furthermore, the two ilia bones from each rat were isolated then one of them was fixed in10 % formalin for histological studies, while the other one and the collected sera were kept at -20 ^o^C for further biochemical analyses.

*Serum calcium concentration*

Diluted serum sample was introduced to an atomic absorption spectroscopy (Perkin-Elmer, Germany) flame where the calcium in its energized form absorbed radiation at 423 m (17).

*Bone calcium amount*

Bones were crushed and dried in an oven at 110 ^o^C for 2 h. The dried powdered bones were mixed with acid solution (H_2_SO_4_ and HNO_3_, 1:1), covered and stored at 37 ^ᵒ^C for one week. The samples were diluted with deionized water and centrifuged at 3000 rpm for 15 min. The precipitate was ashen for 2 h at 500 ^ᵒ^C and the amount of bone calcium was then calculated as follows (18, 19).

$\mathrm{Weight of calcium}(g/\mathrm{g dry bone})=\frac{\mathrm{W.CaS}O_{4}\mathrm{xMr Ca}}{\mathrm{Mr CaS}O_{4}}$

Percentage of iliac bone calcium bone/ iliac bone weight= (weight of calcium presence in one iliac bone (mg)/ weight of the same iliac bone)*100

*Serum inorganic phosphate concentration*

800 µL of blank reagent (H_2_SO_4_, 0.36 M and NaCl, 154 mM) were added to 40 µL serum. Then 400 µL of phosphorus reagent (ammonium molybdate, 3.5 mM, H_2_SO_4_, 0.36 mM and NaCl, 154 mM) were added and incubated for five minutes at 37 ºC. The absorbances of standard and test were measured at 340 nm against blank (20).

Serum alkaline phosphatase (ALP) was carried out according to manufacturer's instruction (21). 

*Serum estrogen and PTH concentrations*

The estrogen and PTH levels was estimated by the enzyme- linked- immunosorbent assay (ELISA) according to manufacturer's guidelines (Calbiotech, Spring Valley, CA) (22) and (23). 

Serum ALT (24), AST (24), Urea (25) and creatinine (26) were measured according to manufacturer's instruction**s.**

*Histological techniques*

*Staining of bone cells*

The fixed iliac bones were transferred to 95 % ethanol for 24 h, decalcified in 7 % nitric acid for about five days at 37 ᵒC, washed for 24 h under running tap water, then soaked in 5 % Na_2_SO_4_. Bones were dehydrated in 95% ethanol then embedded in paraffin. Three 5-mm-thick paraffin-embedded horizontal bone sections were cut from the proximal end of the diaphysis, stained with haematoxylin–eosin and examined by light microscopy (27).

Staining of the Amount of Calcium Deposition in Bone**: **the paraffin sections were placed in 0.5 % aqueous silver nitrate and then exposed to strong light for 1 h. The sections were washed well with distilled water then treated with sodium thiosulfate for 5 min. Sections were washed under running water for 5 min. and counter stained in hematoxylin**, **then passed on 70, 95, 100 % ethanol and cleared then mounted under light microscope (28).

*Statistical analyses*

Data were analyzed by one-way analysis of variance (ANOVA) using Primer of Biostatistics (Version 5) software program. Significance of means ± SD was detected groups by the multiple comparisons Student-Newman-keuls test at p < 0.05.

## Results

The phytochemical characters of moghat are summarized in Table 2. Our qualitative determination shows that moghat contained alkaloids, flavonoids, steroids and cardiac glycoside. On the other side, our quantitative examination proves that* Glossostemon bruguieri* contained 5.8 % alkaloids, 1.5% flavonoids, 13.5% phenols and 4.5 pg/g steroid compounds. Furthermore, each one gram of moghat contained 6.2 mg calcium ions.

**Table 2:** Qualitative and quantitative phytochemical screening of moghat

**Components**	**Presence and concentration** **
Alkaloids	+
Tannins	-
Phlobatannins	-
Saponins	-
Flavonoid	+
Steroids	+
Terpenoid	-
Cardiac glycosides	+
Flavonoid concentration *	1.54 ± 0.05
Alkaloids concentration *	5.8 ± 0.43
Total phenol *	13.18 ± 2.3
Calcium (mg/g root)	6.2 ± 0.1
steroid compound (pg/g)	4.5 ± 0.3

* Values are expressed as % of dry raw weight

** Data expressed as Mean ± standard division "SD", p<0.001

In order to determine the safe medicinal dose of aqueous moghat suspension, acute and sub-chronic toxicity were preformed. Acute toxicity study gives indication about LD_50_ which was higher than 10 g/Kg. In the other side, sub-chronic toxicity is the gate to assess the extract safety, our data considered the moghat safety mentioned in Table 3 shows that the dose of 200, 500 and 1000 mg/mL had no hepato- and/ or nephro-toxicity because the liver and kidney parameters levels were similar to control levels (zero concentration). Otherwise, the dose of 2000 mg/mL showed slightly hepato- and nephro- toxicity.

**Table 3:** The effect of different aqueous suspension of moghat doses on mice physiological parameters.

**Doses / parameters**	**Zero **	**200**	**500**	**1000**	**2000**
Urea (mg/dL)	25.2 ± 0.05 a	26.4 ± 1.0a	26.1 ± 1.5a	27.7 ± 1.6 a	26.6 ± 1.8 a
Creatinine (mg/dL)	0.3 ± 0.02 a	0.35 ± 0.0 a	0.4 ± 0.02 a	0.32 ± 0.1 a	0.8 ± 0.03 b
AST (IU)	13.0 ± 2.7 a	14.1 ± 1.1 a	15.1 ± 2.1 a	14.5 ±1.2 a	20 ± 1.7 b
ALT (IU)	14.0 ± 2.5 a	16.3 ± 0.9 a	15.3 ± 0.9 a	14.6 ± 2.3 a	23 ± 2.9 b
ALP (IU)	63.5 ± 5.5 a	65.3 ± 3.5 a	70.4 ± 8.1 a	60.5 ± 3.2 a	90.9 ± 9.4 b

10 mice in each group were orally administrated 0.2 mL water (zero) or 0.2 mL aqueous suspension of moghat at dose of 200, 500, 1000 or 2000 mg/Kg

Within the row, means with different letters (a or b) were significantly different at p < 0.05. Mean with letter (a) was significantly the lowest value while mean with the letter (b) was significantly the highest value. If two or three groups have the same letters that means there is no significant difference detected at p < 0.05.

**Figure 1:**
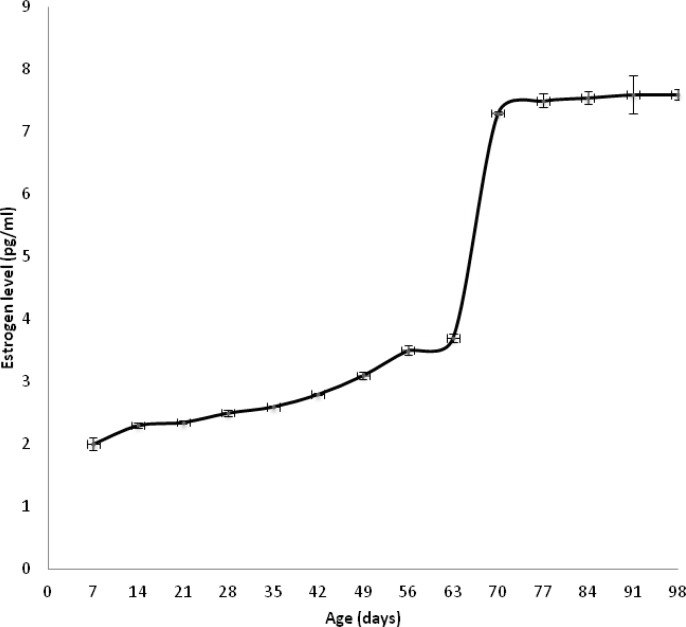
Normal female rat's estrogen level as a function of age

Serum estrogen level was measured as function of age to determine the puberty time for female rats. Figure 1 represents that estrogen level increased slowly and gradually until 63 days old then abruptly. Our data shows that the puberty estimated time was about 70 days.

* Each point is the mean of six determinations ± SD, at p < 0.05.

Free Ca and vitamin D synthetic diet was successfully induced alteration in bone mineralization and body parameters where the body, bone and bone calcium weights were decreased by 59, 55 and 94%, respectively after 21 days induction period and by 35, 37 and 83.6%, respectively after 42 days induction period than that of control groups at p < 0.05. Furthermore, the percentage of bone calcium/bone weight was decreased by 84 and 76% after 21 and 42 days of synthetic diet intake than the normal control ratio. 

**Figure 2:**
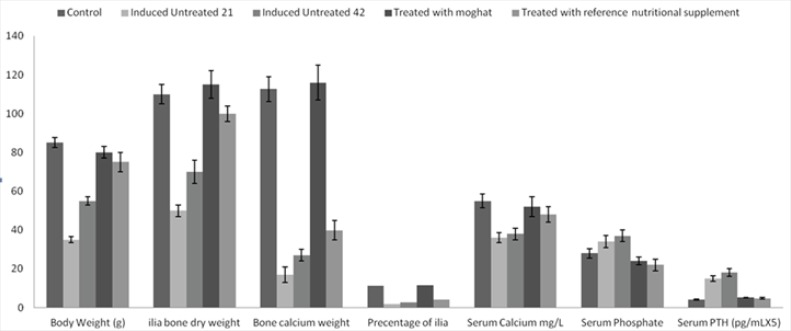
The effects of free calcium and vitamin D- synthetic diet and the treatment of JO with moghat or reference nutritional supplement on weight of body, ilia bone, ilia bone calcium, percentage of ilia bone weight/ilia bone calcium weight, serum calcium, phosphate and PTH levels during juvenile osteoporosis development

Moreover, diet intake for 21 days decreased serum calcium level by 35 % that was combined with a moderate elevation in serum phosphate level by 21 % and extensive elevation in serum PTH by 266 % than those of control group. On the other hand, 42 days diet intake reduced the serum calcium by 31% and elevated both serum phosphate and PTH levels by 32 and 339 %, respectively than those of the control one, at p < 0.05 (Figure 2).

**Figure 3:**
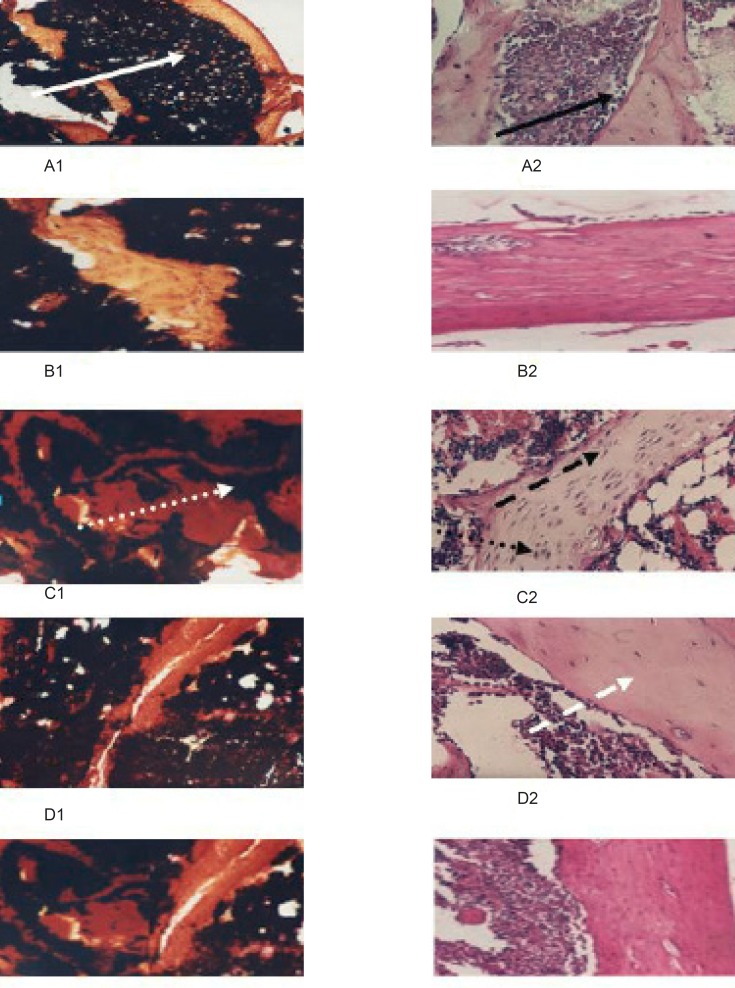
Effect of the treatment by Moghat on calcium deposition of IJO induced rat.

The histological studies (Figure 3) shows that free Ca-vitamin D synthetic diet intake either for 21 days, or 42 days, decreased the amount of deposited calcium (black spots, B1 and C1, respectively) than that was represented in the control group (A1). This was combined with hyperactivity in osteoblastic cell associated with the elevation in the number of osteoclastic cell and lowering in collagen content (B2 and C2, respectively) (indicating by the intensity of stain) when compared with control group (A2).

Diet intake lead to sever alteration on liver and kidney functions parameters as shown in Table 4 where AST, ALT, ALP, urea and creatinine were increased by 3, 1.2, 3.5, 1.3 and 1.6 fold after 21 days induction period than those of control group and by 3.9, 1.2, 4.7, 1.3 and 1.7 fold after 42 days induction period than those of control levels. 

Treatment with moghat suspension at a dose of 0.8 g/k/day successfully alleviated the body mass, ilia bone and bone calcium weights to normal control value, at p < 0.05 (Figure 2). Furthermore, moghat treatment increased serum calcium level 37% over the induced untreated 42 level and decreased serum phosphate and PTH levels by 46 and 68 % respectively than that of the corresponding induced untreated 42 levels, at p < 0.05 (Figure 2).

These results were confirmed by the histological studies (Figure 3) which shows that moghat intake for 21 days increased the amount of deposited calcium (black spots, D1) than that represented in the induced untreated 42 group (C1) that was combined with norm-osteoblastic cells (D2) when compared with control group (A2) and the hyper-activated one in the induced untreated 42 group (C2).

Von-Kossa staining shows the calcium deposition in a longitudinal section at magnification 100x of light microscope for (A1) Control female rat (B1) Induced untreated 21-female rat (C1) Induced untreated 42-female rat (D1) Moghat-treated female rat (E1) Treated with reference nutritional supplement.

Effect of the treatment by moghat on bone cells of IJO induced rat.

Hematoxylin-eosin staining shows the cells in a longitudinal section at magnification 400x of light microscope for (A2) Control (B2) Induced untreated 21-female rat (C2) Induced-untreated 42- female rat (D2) Moghat -treated female rat (E2) Treated with reference nutritional supplement.

Solid white arrow: Normal Deposited calcium --- Solid black arrow: Normal Osteoblast --Dashed white arrow: Normal Collagen Stain--- Dashed black arrow: Hyper-activated Osteoblast --- Doted white arrow: Low Calcium Deposited --- Doted black arrow: Osteoclast cells.

The same biochemical (Table 4) and histological (Figure 3 E1 and E2) patterns were observed for the treatment with the reference nutritional supplement. Otherwise, treatment with moghat is more potent than that displayed with the reference nutritional supplement.

The estrogen levels of JO-induced, moghat treated and reference nutritional supplement treated groups were similar to control estrogen level, at p < 0.05 (Table 4).

Urea level was significantly increased in rat received moghat treatment by 40%, while creatinine level was decreased by 16% in rat received reference nutritional supplement than those of the induced untreated 42 levels at p < 0.05 (Table 4). Unfortunately, both treatment strategies failed to normalize kidney function parameters, at p < 0.05.

Both treatment regiments successfully normalized liver function parameters where they decreased the AST, ALT and ALP levels by 81, 41 and 81 % in moghat regiment and 91, 47 and 50 % in reference nutritional supplement regiment, respectively than those showed in the induced untreated 42 group, at p < 0.05, Table 4. 

**Table 4:** The effects of the treatment of juvenile osteoporosis female rats by moghat and reference nutritional supplement on body weight and osteoporotic markers

**Groups/parameters**	**Control **	**Induced untreated 21 **	**Induced untreated 42**	**Treated with moghat **	**Treated with reference nutritional supplement**
Estrogen (pg/mL)	3.2 ± 0.1 b	2.5 ± 0.01 a	3.1 ± 0.01 b	3.1 ± 0.01 b	3.2 ± 0.19 b
Urea (mg/dL)	21.1 ± 1.0 a	26.4 ± 1.0b	26.1 ± 1.5b	36.7 ± 1.6 c	26.6 ± 1.8 b
Creatinine (mg/dL)	0.7 ±0.0 a	1.1 ± 0.0 b	1.2 ± 0.2 b	1.0 ± 0.1 b	0.8 ± 0.1 a
AST (IU)	23.0 ±1.7 c	70.1 ± 9.1 d	90.1 ± 12.1 e	17.5 ± 3.2 b	8.6 ± 10.7 a
ALT (IU)	22.0 ± 1.5 b	26.0 ± 2.1 c	28.0 ± 0.9 c	16.5 ± 2.3 a	15.3± 1.9 a
ALP (IU)	65.5 ±3.5 a	227.3 ±14.5 c	312.1 ± 10.1 d	60.5 ± 7.8 a	160.9 ± 9.4 b

Control; Animal fed regular pelleted food, Induced-untreated 21; Animal fed Ca^2+ ^and vitamin D-free-synthetic diet for 21 days. Induced-untreated 42; Animal fed Ca^2+ ^and vitamin D-free-synthetic diet for 42 days, Treated with reference nutritional supplements; Animal fed Ca^2+^-synthetic diet and treated with a combination of 750 µM Ca^2+^, 1 µM and 2 IU vitamin D_3_, Treated with moghat; Animal fed Ca^2+^-synthetic diet and treated with 0.8 g/Kg moghat

Within the row, means with different letters (a, b, c, d or e) were significantly different at p<0.05. Mean with letter (a) was significantly the lowest value while mean with the letter (e) was significantly the highest value. Letters b within the row is significantly different from a and c and so on. If two or three groups have the same letters that means there is no significant difference detected at p < 0.05.

## Discussion

The World Health Organization (WHO) estimates that herbal medicine care is about 75-80% of the world's population, mainly in the developing countries, because of better cultural acceptability, better compatibility with the human body and fewer side effects (29).

In Egypt, moghat root powder is used as a tonic, nutritive and gout curative agent due to its highly mucilage content, furthermore it is prescribed as a demulcent agent (30). Several plants that are rich in alkaloids, flavonoids, tannins and saponins exhibit medicinal and physiological activities (31). There are few studies considered moghat secondary metabolite content but it was established that moghat contains phytosterols, biflavone and other compounds such as sesamin, emodin, chrysophanol and physcion (32). Beside these findings, our results represented that moghat could be considered as a rich source for steroids, cardiac glycosides, alkaloid , flavonoid , estrogenic compounds, total phenol and calcium ions(Table 2). Furthermore, moghat is safe medicinal plant as our results indicated that sub-chronic toxicity occurred at dose of 2000 mg/Kg (Table 3) and the LD50 dose is higher than 10 g/Kg. In agreement with our results, it was found that moghat powder was proved to be non-toxic up to 2.5 g/Kg body weight; also no side effect of moghat intake was reported in liver and kidney functions (31).

Plants derived phenolic compounds manifested beneficial effects and potential inhibition of several stages carcinogenesis and several serious diseases (33). Furthermore, phytoestrogens are a group of biologically active plant substances with a chemical structure similar to that of estradiol, an endogenous estrogen. This structural similarity accounts for the ability of these compounds to bind to estrogen receptor and exert various estrogenic or antiestrogenic effects (33). There are three main classes of phytoestrogens: isoflavones, coumestans, and lignans, which occur in either plants or seeds (33). Several flavoind and alkaloids used in the treatment of several diseases such as osteoporosis. Where, it is recently found that the flavonoids, isoflavonid and alkaloids intake showed an anabolic effect on bone component through the potent inhibitory effect on osteoclastogenesis and bone resorption rather than bone formation (34, 35). Recently, sesemin, one of moghat active ingredients (32), was being more promising than amine in dental resin formulations because it has a co-inhibitory effect for dental resorption (36). 

Based on our phytochemical results, we speculated that moghat could be used as vital nutritional and medicinal agents due to the presence of estrogenic like compounds, alkaloid and flavoind. Furthermore, as it contains isoflavonid and sesemin, it could be a good nutritional reversal therapy for osteopenia. Osteopenia or bone mass loss is due to increased bone resorption and decreased bone formation (37). 

The two obstacles in this study were the lack of an animal model to study JO and the natural reversal therapeutic effect of ovarian estrogen during the onset of puberty. Unfortunately, the successful reported procedures to induce osteoporosis are ovariectomization and corticosteroids administration (38) that produced simulator adult human osteoporotic animal model. To resolve these problems, we firstly determined the approximate puberty age which was 70 days. Therefore, we started the JO induction protocol by feeding 15 day old female rat’s free Ca and vitamin D synthetic diet rich in sodium, phosphorus and protein for 21 days. After induction period, two treatment strategies were carried out for extra 21 days; therefore, our experimental treatment protocol was ended at the age of 57 days which was prior the puberty time onset by 13 days. Altogether, this design emphasized that any obtained ameliorated reversal effects toward JO could be returned to our treatment protocols not to naturally estrogen therapeutic effect. 

The design of the diet based on the facts that vitamin D_3_ and calcium deficiency shall lead to rickets and bone mass loss, respectively (3,4). High sodium intake is associated with reduced bone mineralization at the hip (5). Furthermore, excess dietary phosphate is attributed to a secondary hyperparathyroidism leading to bone loss disorder (3, 39).

Our results showed that, synthetic diet intake for 21 days or 42 days lead to loss or normal walk ability, aggressive behavior incidence (observation), body mass loss, bone and bone calcium masses reduction as shown in Figure 2. Furthermore, These findings were supported by histological study that revealed a serious bone loss indicated low deposited calcium and collagen that associated with the spindle shape of osteoblastic cells that revealed cellular hyperactivity o Moreover, our data showed that hypocalcaemia and hyperphosphatemia were took placed in association with hyperparathyroidism linked with high serum ALP activity. 

Body mass loss could be attributed to the loss in the appetite which resulted from low calcium intake (3). Low calcium intake lead to alteration in gut calcium absorption which in turn disturbs calcium homeostasis leading to an imbalance in the calcium regulating hormones (parathyroid hormone and calcitonin) and thereby increases bone turnover (40). Bone turnover is characterized by losing of both bone protein and mineral (41) associated with high osteoclasts formation, osteoblast hyperactivity and high blood ALP activity (42). So we concluded that osteopenia took place in our rat model due to hyperparathyroidism that resin from imbalance diet. Our data showed that, AST, ALT, creatinine and urea (Table 4) were increased. Hyperparathyroidism is contributed as a major factor in chronic renal failure incidence due to phosphorus retention and hypocalcaemia which accelerated the kidney stone formation that is diagnosed with elevation of both blood AST activity, urea and creatinine level (43). Altogether, it is clear that we succeeded to design an appropriate valid JO animal model with high similarities to human in their pathophysiological mechanisms of bone deterioration.

The treatment with reference nutritional supplement or moghat was successfully returning the body and bone masses back to the control values, furthermore, no side effects on liver was detected. So, the treatment seemed sufficient to treat bone loss.

The combination of Ca and vitamin D_3 _normalized the ALP activity, serum levels of calcium, phosphate and PTH and increased calcium mass by 1.5 fold, but did not return it back to the control value. It was reported that treatment with calcium and vitamin D was able to ameliorate bone loss but unable to normalize the bone density (44).

Treatment with moghat suspension normalized serum ALP activity, calcium, phosphate and PTH levels and elevated the bone and calcium masses higher than control values. Furthermore, the osteoblast hyperactivity was disappeared after moghat extract co-administration. These findings showed the positive and unique role of moghat in stimulating osteoblasts while also damping osteoclasts. However, the basis of the dual activity was unknown. Therefore, these results strongly suggested that moghat might be a new therapy for the management of JO. This effect was mainly due to the presence of phytoestrogen that acted on estrogenic receptors on bone and therefore increased the bone formation.

In conclusion, the use of free calcium and vitamin D synthetic diet to induce JO in experimental animals is a good and precise protocol and gives an excellent simulator model for human JO. Moghat showed a positive effect on osteoporotic bone in the present of valid JO experimental animal model confirmed by decreasing osteoclastic resorption and increasing osteoblastic formation markers. Collectively, our results strongly suggest that moghat might be a new potential therapy for the management of JO in humans combining a powerful bone-forming as well as an anti-resorptive activity.

## Conflict of interest statement

We declare that there is no disclose any financial and personal relationships with other people or organizations that could inappropriately influence (bias) our work.
